# Redox Properties of Tryptophan Metabolism and the Concept of Tryptophan Use in Pregnancy

**DOI:** 10.3390/ijms18071595

**Published:** 2017-07-24

**Authors:** Kang Xu, Hongnan Liu, Miaomiao Bai, Jing Gao, Xin Wu, Yulong Yin

**Affiliations:** 1 Chinese Academy of Sciences, Institute of Subtropical Agriculture, Key Laboratory of Agroecological Processes in Subtropical Region, Changsha 410125, China; xukang2020@163.com (K.X.); liuhn@isa.ac.cn (H.L.); miaomiaobai1115@126.com (M.B.); gaojing.he@163.com (J.G.); wu-xin@foxmail.com (X.W.); 2 National Engineering Laboratory for Pollution Control and Waste Utilization in Livestock and Poultry Production, Changsha 410125, China; 3 Hunan Provincial Engineering Research Center for Healthy Livestock and Poultry Production, Changsha 410125, China; 4 Scientific Observing and Experimental Station of Animal Nutrition and Feed Science in South Central, Ministry of Agriculture, Changsha 410125, China

**Keywords:** tryptophan, antioxidant, oxidative stress, pregnancy, tryptophan metabolites

## Abstract

During pregnancy, tryptophan (Trp) is required for several purposes, and Trp metabolism varies over time in the mother and fetus. Increased oxidative stress (OS) with high metabolic, energy and oxygen demands during normal pregnancy or in pregnancy-associated disorders has been reported. Taking the antioxidant properties of Trp and its metabolites into consideration, we made four hypotheses. First, the use of Trp and its metabolites is optional based on their antioxidant properties during pregnancy. Second, dynamic Trp metabolism is an accommodation mechanism in response to OS. Third, regulation of Trp metabolism could be used to control/attenuate OS according to variations in Trp metabolism during pregnancy. Fourth, OS-mediated injury could be alleviated by regulation of Trp metabolism in pregnancy-associated disorders. Future studies in normal/abnormal pregnancies and in associated disorders should include measurements of free Trp, total Trp, Trp metabolites, and activities of Trp-degrading enzymes in plasma. Abnormal pregnancies and some associated disorders may be associated with disordered Trp metabolism related to OS. Mounting evidence suggests that the investigation of the use of Trp and its metabolites in pregnancy will be meanful.

## 1. Introduction

Pregnancy can be characterized by increased basal oxygen consumption and a state of high oxidative stress (OS) [1]. Undesirable pregnancy outcomes can be induced by uncontrolled OS. Maternal OS or low intake of antioxidants are involved in some disorders during pregnancy, such as preeclampsia, shortened duration of gestation, low birth weight, and minor congenital defects [2,3], and have been suggested to be antecedent to the pathophysiology of adverse outcomes rather than the consequence [4]. Hence, establishment of an appropriate antioxidant status to prevent OS is more effective than using antioxidants after adverse effects occur during pregnancy. Effective antioxidants and reasonable supplementation are important for maintaining an appropriate antioxidant status.

Tryptophan (Trp) is an essential amino acid and has an important role in protein synthesis, but a low proportion (<1%) is used for this. In addition, Trp and its metabolites have important roles in other biologic functions, including the generation of 5-hydroxytryptamine (5-HT), melatonin and other bioactive molecules. Trp metabolites containing 5-HT, melatonin, kynurenic acid (KYNA), nicotinamide adenine dinucleotide (NAD), nicotinamide adenine dinucleotide phosphate (NADP) and others are essential for normal metabolism and organ functions [5]. Trp and some metabolites (melatonin, KYNA and xanthurenic acid (XA)) may act as antioxidants that can remove reactive oxygen species (ROS) effectively and enhance resistance of the damage caused by free radicals [6,7,8].

Studies have focused on the evaluation of OS in pregnancy, the antioxidant properties of Trp and its metabolites, and Trp metabolism during normal and abnormal pregnancies. Herein, we review evidence to identify the underlying correlation between Trp metabolism and OS during gestation, how it may help to attenuate OS through modification of the intake and metabolism of Trp, and how feeding strategies can be adjusted and chosen according to the redox states to enhance an appropriate antioxidant status during gestation.

## 2. OS during a Normal Pregnancy

Increased OS had been reported to be associated with high metabolic demand, energy demand, and requirements for tissue oxygen during normal pregnancy in humans [9,10], sows [11], dogs [12], sheep [13] and other species [9]. Uncontrolled OS during pregnancy may result in embryo resorption, placental degeneration, restriction of fetal growth, pregnancy interruption, stillbirths, and other problems [13]. A low antioxidant capacity of embryos and a relatively low oxygen environment is needed for embryonic development, and the embryo is sensitive to oxidant molecules [14]. In animal models, OS has been shown to influence pregnancy outcomes [4]. In sows, increased OS can alter formation of the placenta and fetal skeleton [15], negatively affect the growth and health of fetuses, and be responsible for reduced reproductive performance, milk production, and longevity [16]. During pregnancy in rats, reduced placental perfusion is associated with OS in intrauterine fetal growth restriction [17].

Imbalance between the production of reactive species (ROS, reactive nitrogen species (RNS) and others) and antioxidant capacity is a direct cause of OS. The primary reason for high OS during pregnancy is an important parameter. During pregnancy, a drastic increase in energetic and nutritional demands is needed to ensure adequate placental formation, blastocyst implantation, as well as fetal development and growth. This is followed by a sharp alteration in metabolic equilibria, upregulation of energy metabolism [18], increased synthesis of adenosine triphosphate and a high requirement of irrigation and oxygenation for tissues (mostly, the placenta and fetus) [13]. All of these changes lead to over-generation of ROS in placental, embryo and fetal mitochondria, which then make mother and fetus more likely to experience OS [13]. 

Pregnancy is considered to favor OS, and several factors trigger generation of ROS and OS [19]. An increased number of mitochondria and a high metabolic rate in the placenta, and an increased partial pressure of oxygen (pO_2_) of blood in the mother have been recognized as major factors [20]. From early pregnancy, the placenta, a source of antioxidative enzymes and hormones to control placental lipid peroxidation (LP) [21], has a major influence on fetal and maternal homeostasis [19]. The requirement of fetal development and growth leads to an accelerated transplacental exchange of nutrients, and is accompanied by increased numbers of placental and fetal mitochondria as well as high placental metabolism and steroidogenesis [13,22,23]. Rich in mitochondria, the placenta consumes 1% of the maternal basal metabolic rate when fully developed [24]. This high metabolic demand may induce placental production of ROS [25,26]. Macrophages, which favor the local placental production of free radicals, are also rich in the placenta [19]. High concentrations of malondialdehyde (MDA) have also been detected in placentomes [27].

During pregnancy, another major factor involved in ROS generation is a variation in the pO_2_ of blood [19]. Initially, before the development of blood vessels and maturation of the placenta, the latter is in a hypoxic environment. A lower pO_2_ exists in the placental cellular structure and fetal circulation, and induces a greater affinity for oxygen. Hence, the oxygen released from maternal hemoglobin^4^ (Hb)^4^ is favored by the placenta. Meanwhile, with the release of fetal and placental metabolites (such as lactic acid), the pH of blood is lowered, which favors oxygen delivery from Hb [19]. As the placenta changes to become an oxygen-rich environment with abundant mitochondria, ROS production in the placenta is increased, and induces adverse effects in the placenta, fetus, and mother [19,28]. During twin pregnancies, a higher oxygen requirement and more intense LP inducesmore obvious adverse effects from ROS. In addition, the MDA level increases in proportion to the number of fetuses in multiple births [29].

In addition, an increased systemic OS during late gestation and lactation has been reported in highly prolific animals, such as sows [11]. Oxidative damage to protein and DNA are increased further during late gestation and lactation due to a severe catabolic status and enhanced production of ROS by the placenta [11,30]. Plasma concentrations of vitamin E and retinoid have been reported to decrease by 56% and 57% at day 110 of gestation compared with day 30 of gestation, respectively. During late gestation, endogenous damage to the DNA of lymphocytes has been reported to increase by 125% compared with that at day 30 of pregnancy, thereby suggesting increased damage to immune cells [30].

## 3. The Defense Strategy against OS in Pregnancy

Before or during gestation, an appropriate antioxidant status is important for placental development, a reduction in the risk of embryo mortality, an improvement of outcome, and the vitality of the newborn [13]. Adequate energy, mineral, vitamin and antioxidant supports aid the efficacy of antioxidant enzymes and provide good defense potential, which enhances maternal and fetal protection [13]. Antioxidant supplementation is important for maintaining cellular redox status, protecting enzymes and proteins, and inhibiting peroxidation during a normal pregnancy. Reasonable intake of antioxidants is important for maintaining an appropriate antioxidant status.

At present, vitamins C and E are usually chosen as antioxidants during pregnancy. Vitamin C can scavenge free radicals in the aqueous phase, vitamin E can prevent LP in vivo, and both can protect against OS. These antioxidants could improve OS, and reduce the risk of the pregnancy disorders associated with OS, such as abruptio placentae and preeclampsia with a low baseline antioxidant status [31,32]. However, these antioxidants have certain limitations. Firstly, in some conditions, supplementation with vitamin C and vitamin E cannot reduce the risk of preeclampsia [32], prevent preeclampsia in women at risk [33], or reduce the pregnancy-associated hypertension [34]; Secondly, supplementation with vitamin C and E during pregnancy has been associated with an increased risk of gestational hypertension, higher stillbirth rate, premature rupture of membranes, and an increased prevalence of babies born with a low birth weight [32,33,34].

Why do invalid, even harmful, effects appeared when supplementation with vitamin C and vitamin E during pregnancy? First, the above physiological doses of vitamins supplementation may be one factor. In these experiments, the baseline antioxidant status were no assessed. The supplementation of antioxidants may induce the obstructed free radical formation, and cause disrupted ROS homeostasis, then induce side effects of pregnancy; Second, no apparent benefit of vitamin C and E supplementation was detected, whether it is just limitation of these two vitamins or all of antioxidant, the effectiveness evaluation for other antioxidants would be necessary during pregnancy. Research have hypothesized that non-antioxidant effects of exogenous vitamin E may cause detrimental effects on human pregnancy; Third, the undefined pathophysiology of some pregnancy disorders need attentions. For details, maybe OS is present in preeclampsia, but it is not important in the pathophysiology. OS may even play a major role in the pathophysiology, but it is not dominant on causing disorder. Alternatively, OS may just be relevant to the pathogenesis of some pregnancy disorders in only a subgroup, with no appreciable benefit of vitamins C and E for the entire population. For these, to better and security use antioxidant during pregnancy is challenging.

Firstly, before use antioxidants, specific quantitative indices of OS (such as LP and antioxidant status) could be considered as entry criteria in clinical trials during pregnancy. This may be a guide to decide whether to add and dosage of supplementation antioxidants. In addition, more unconventional indices of OS, especially endogenous metabolites with redox regulation property, should be screened and applied, which is beneficial for the evaluation of baseline antioxidant status during pregnancy; Secondly, the non-antioxidant effects of exogenous antioxidants should be assessed during pregnancy, because there is limited evidence on assessing their safety. More trial about the safety of use during pregnancy of antioxidants that contain vitamin C and vitamin E should be inevitably raised. In the same time, the long term effects of antioxidants on both women and children should be determined for supplementation during pregnancy; Thirdly, the underlying pathophysiology should be further considered. In the same time, the effect of the antioxidant intervention on markers of OS and placental function should be investigated.

Hence, studies are essential to assess the application of endogenous indices of OS, the effects of new, specific antioxidants and patterns of dietary supplementation during pregnancy. Because of the potential antioxidant capacity of Trp and its metabolites, the nutritional functions of Trp, and the variation and abnormal Trp metabolism induced by OS during pregnancy, new strategies are available for regulating Trp metabolism and Trp addition to lower the risk of OS during pregnancy. Such strategies are discussed below.

## 4. Trp Metabolism and Antioxidants

**Figure 1:**
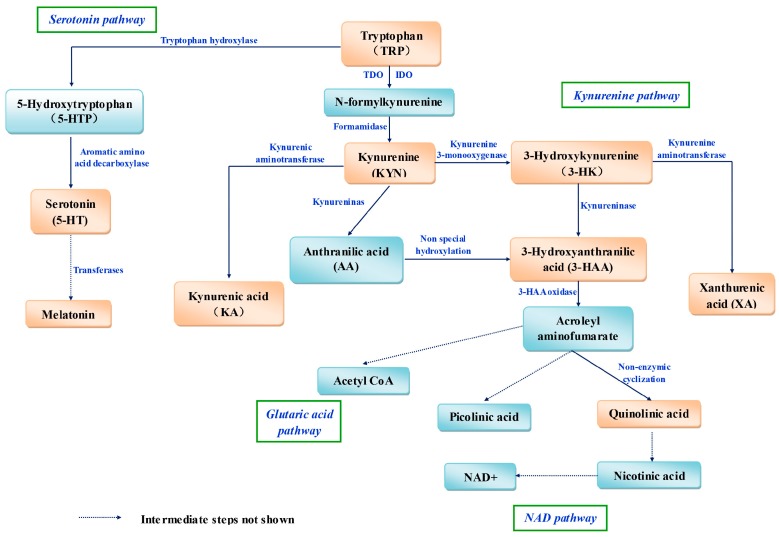
Schematic diagram of tryptophan metabolism in mammals. Several intermediate metabolites and enzymes are shown; Some metabolites with redox properties are in orangered frames. NAD, nicotinamide adenine dinucleotide; NAD^+^, oxidized form of nicotinamide adenine dinucleotide; Acetyl CoA, acetyl coenzyme A.

In mammals, most dietary Trp is metabolized via four pathways (Figure 1). The hepatic (oxidative) kynurenine pathway (KP) is the most important because 95% of Trp is degraded through it [35], and Trp 2,3-dioxygenase (TDO) is the rate-limiting enzyme. The reaction intermediates and enzymes in the kynurenine pathway (KP) are outlined in Figure 1. The KP is also present in extrahepatic tissues, but it is regulated mainly by another rate-limiting enzyme, indoleamine 2,3-dioxygenase (IDO), the activity of which is 5–15% that of hepatic TDO [36].

When evaluating the antioxidant properties of Trp metabolism, one should also consider Trp and all of its metabolites. First, one should pay attention to melatonin. In several studies, melatonin has been shown to be a highly efficient antioxidant in vivo and in vitro. However, the transformation efficiency of Trp to melatonin is low, and the interrelationship between oral feeding of Trp and melatonin concentration in organisms should be studied.

Various Trp metabolites are derived from the KP [37,38]. Kynurenine (KYN) [39], KYNA, 3-hydroxyanthranilic acid (3HAA) [7], 3-hydroxykynurenine (3-HK) [7] and XA [7,40] can act as potent scavengers of free radicals in organisms. The rate-limiting enzyme IDO has also been shown to be a “waste collector” to “clean up” free radicals in the body [41]. IDO appears to be a more efficient ROS scavenger than superoxide dismutase (SOD), which suggests that the high activity of IDO might represent one way in which the body “dampens” the effects of OS [41].

However, on the contrary, Trp and its metabolites containing KYN, KYNA, and 3HAA have also shown pro-oxidant effects [42,43]. The activation of the kynurenine pathway was proposed to produce oxidative stress. Some of the kynurenine pathway metabolites (3-HK [44], 3HAA, and quinolinic acid (QUIN) [45]) can generate free radicals under physiological conditions [45], and lead to cellular oxidative stress and damage.

### 4.1. Tryptophan

The antioxidant effects of Trp have been reported in some foods and animal-feed additives. In eggs [46], bacon [47], potatoes [48] and egg tofu, Trp had been shown to be an important antioxidant. In these foods, a positive correlation between the content and antioxidant capacity of Trp has been detected. Recently, researchers discovered the antioxidant properties of Trp in human breast milk, and showed that Trp can remove free radicals effectively [49].

In an in vitro experiment using human glioma cells, Trp was shown to have strong antioxidative properties [50]. In rats under endotoxic shock, Trp was demonstrated to be an effective scavenger of free radicals, and to reduce the damage caused by free radicals to organs and cells [51]. In extracts of human placenta, l-Trp was shown to be the main antioxidant and had higher suppressive activity against cytochrome P-450-dependent LP in OS [52]. In addition, in the placenta from a woman suffering from preeclampsia, Trp catabolism and levels of its related metabolic enzymes were lower than that from normal volunteers. Animal experiments have shown that Trp can reduce the prevalence of miscarriage and mortality of toxicity-induced pregnant mice, and also increase the fetal survival rate and live litter size [53]. The protective effect of Trp may not only be its own redox property, but also due to the Trp metabolites with antioxidant properties [54]. Evidence regarding the antioxidant effects of Trp has been reported, but the mechanism of action has not.

However, in another research, pro-oxidant effect had been detected after oral tryptophan. Each 6 g oral Trp loading can induced an increase in lipid peroxidation products and kynurenines in blood from 15 healthy volunteers [42]. The oxidative stress caused by Trp loading was suggested to result from the generation of 3-HK, 3HAA and QUIN, all of which can generate free radicals [42]. Morever, another research showed that loading of up to 5.0 g/day l-Trp had no effect on mood states or other adverse effects in healthy women. However, the urinary excretion of Trp metabolites, including KYN, AA, KYNA, 3-HK, 3HAA, and QUIN, was in proportion to l-Trp loading [55]. The conflicting result compared with above studies may be caused by the different intake Trp content, the different status of experiment subjects or the different timepoints after Trp loading. Many influenced factors and caution should be exercised in the use of Trp loading, which can remove free radicals effectively or induce OS.

### 4.2. Kynurenine

Kynurenine (KYN) is a central compound of the KP, and can scavenge intracellular ROS and other free radicals [39]. In vitro studies have shown that KYNA has scavenging properties. Kynurenine can donate an electron and protect macromolecules in vivo and in vitro against oxidative modifications, but its scavenging activity is obviously lower than that of other KP metabolites, such as KYNA and XA [7,56]. Hence, the direct scavenging activity of kynurenine is not the primary manner by which it acts in vivo. 

KYN can scavenge in two main ways in an organism. First, scavenging activity can be mainly by KYNA production. KYNA can scavenge hydroxyl radicals (^•^OH) efficiently and prevent LP and ROS production [57]. Second, kynurenine can inhibit ROS production through a phagocytic defect or inactivation of nicotinamide adenine dinucleotide phosphate (NADPH) oxidase: an increase in kynurenine levels leads to a proportionate decrease in ROS production; an absence of kynurenine induces ROS production in vivo [39].

However, KYN can also acts as a pro-oxidant. Aerobic irradiation of KYN can induce superoxide radicals and leads to reduction of cytochrome c [43]. Additionally, KYN is able to photooxidize cysteine, NADH, and ascorbic acid in vitro studies. In addition, KYN can induce apoptosis mediated by ROS in NK cells [58].

### 4.3. Kynurenic Acid

Kynurenic acid (KYNA) is a major metabolite of the KP. KYNA synthesis is catalyzed by kynurenine aminotransferases (KATs), and KYNA is irreversibly derived from kynurenine in the brain and peripheral tissues [37]. In numerous studies, KYNA has been demonstrated to be a regulator of antioxidative stress [57,59] and anti-inflammatory effects [60] in vivo and in vitro. The antioxidant properties of KYNA can be divided into two aspects. First, due to its redox properties, KYNA can scavenge ^•^OH [43,61] and peroxynitrite [57], and prevent LP, as well as the production of ROS and MDA. Second, by interacting with G protein-coupled receptors, KYNA can modulate the transmission of glutamate and choline, which leads to a reduction in extracellular levels of glutamate and prevents the release of pro-inflammatory cytokines under inflammatory conditions [60]. These two antioxidant features of KYNA are independent of each other.

To explain the redox properties of KYNA, a putative mechanism is that in three steps reaction, KYNA acts as redox catalyst (electron transfer) to catalyze the reaction between two ^•^OH and one superoxide anion (O_2_^•−^), which leads to the release of nitric oxide and carbon dioxide [57,62]. The efficiency of KYNA to scavenge O_2_^•−^ is almost tenfold higher than that of glutathione (GSH) [57]. Taken together, these findings suggest that KYNA may be a highly attractive scavenger.

The antioxidant properties of KYNA in biological systems have been estimated. One in vivo study showed that KYNA mitigates the ^•^OH formation induced by iron (II) sulfate (FeSO_4_) in the rat striatum [57]. Moreover, in *Xenopus laevis* oocytes, a certain concentration of KYNA was shown to attenuate the increased LP and ROS formation caused by FeSO_4_ [57]. Evidence shows that KYNA can act as an important endogenous antioxidant in organisms.

### 4.4. 3-HK

3-HK is another metabolite of the KP. The synthesis of 3-HK is catalyzed by kynurenine 3-monooxygenase, and 3-HK is derived from kynurenine. As the *o*-aminophenol structure in a molecule, 3-HK has pro-oxidant and antioxidant activities [38,43]. With regard to the pro-oxidant properties of 3-HK, researchers have found that in some regions of the brain, a low concentration of 3-HK (1–10 μM) can induce ROS generation. In addition, 3-HK can enhance the activity of endogenous xanthine oxidase by affecting the generation of hydrogen peroxide (H_2_O_2_), which then exacerbates cell damage [63]. However, the precise mechanism by which 3-HK induces ROS and generates H_2_O_2_ in these processes is not clear. However, researches suggested that though 3-HK might has pro-oxidant activities under certain conditions, these actions serve to evoke a redox modulatory activity, and in turn, decrease the odds of cell damage [63].

Conversely, 3-HK has been proposed to be a free radical-scavenger and an antioxidant in some studies. Assays to evaluate the potency of free radical-scavenging based on immunofluorescence or kinetic analyses have shown that 3-HK can scavenge peroxyl radicals with high efficiency. Among Trp metabolites, the free-radical reactivities of 3-HK and 3HAA are equal to or more reactive than those of vitamin C or vitamin E, which are used as efficient free radical-scavengers and antioxidants [7,64]. In addition, when reacted with the ferryl complex, 3-HK was more reactive than GSH, reflecting the higher antioxidative efficiency of 3-HK [65].

The antioxidant properties of 3-HK in biologic systems have also been studied. In the Malpighian tubules of insects, 3-HK is abundant and suggested to be a major antioxidant [66]. Additionally, if exposed to 3-HK, the total antioxidant reactivity of C6 glioma cells was shown to be increased significantly [65]. In the rat striatum, 3-HK has an impact on LP in a concentration-dependent manner: at low micromolar concentrations, it shows its pro-oxidant action; at a higher concentration (100 mM), it presents its antioxidant activity. In addition, 3-HK can stimulate expression of the antioxidant regulator NF-E2-related factor 2 (Nrf2), glutathione *S*-transferase and SOD [63]. Due to these evidences, 3-HK seems to be more a redox modulatory molecule rather than a neurotoxic metabolite. Because of the different roles (pro-oxidant and antioxidant) of 3-HK in the regulation of cellular redox status, when use it, we should consider its concentration, the redox status of cells, and whether cells are involved in inflammatory reactions.

### 4.5. 3-Hydroxyanthranilic Acid

3HAA is an intermediate Trp metabolite along the KP. 3HAA is transformed from 3-HK after catalysis by kynureninase [67]. The antioxidant properties of 3HAA can be ascribed to its electron donation. In chemical assays, 3HAA can scavenge ROS in a concentration-dependent manner [68] and inhibit the oxidation of β-phycoerythrin even more efficiently than other KP metabolites, such as KYNA and XA [7]. Furthermore, 3HAA can scavenge more peroxyl radicals than vitamin C or vitamin E [7]. 

3HAA can act as antioxidant against OS in cells or tissues. In the brain, liver and kidney tissues of rats, 3HAA can attenuate the ROS production, LP and cell dysfunction caused by exposure to pro-oxidants (FeSO_4_ and ONOO−) [68]. Moreover, 3HAA can also prevent LP in the cerebral cortex of rats, and can significantly prevent spontaneous oxidation of Glutathione (GSH) [65]. In murine macrophages stimulated with interferon-γ (IFN-γ) and lipopolysaccharide (LPS), 3HAA downregulates the expression of inducible nitric oxide synthase (iNOS) by affecting hemeoxygenase-1 (HO-1) expression, which is a protective mechanism against OS [69]. Moreover, in human astrocytes, 3HAA can increase the expression of HO-1, an antioxidant enzyme with anti-inflammatory and cytoprotective properties [70]. In human umbilical vein endothelial cells, 3HAA was found to induce HO-1 expression and to stimulate nuclear translocation of Nrf2 [71], which can stimulate the activation of numerous genes encoding antioxidant proteins and anti-inflammatory enzymes [72]. Studies suggest that the 3HAA is a potential antioxidant.

On the contrary, other studies also considered 3HAA as a pro-oxidant due to its auto-oxidation and ability to generate free radicals [43,44,73]. Auto-oxidation of 3HAA requires molecular oxygen and simultaneously generates superoxide radicals and H_2_O_2_ [43]. In cultured neuronal cell, 3HAA could induce protein oxidative damage and apoptosis associated with chromatin condensation and internucleosomal DNA cleavage [74]. In monocyte-derived cells, 3HAA can induce apoptosis and free radical formation, which can be enhanced by addition of ferrous or manganese ions. In addition, the 3HAA induced apoptotic response was slightly attenuated by catalase, which indicated that this response was involved in hydrogen peroxide production [75].

The contradictory redox properties of 3HAA were shown in vivo or vitro. One electrochemical study explained that 3HAA can initially act as an antioxidant and next as a pro-oxidant [76]. However, the most likely explanation for the dual effect in vitro of 3HAA is a concentration-dependent action.

### 4.6. Xanthurenic Acid

The Trp metabolite XA is transformed from 3-HK along the KP. KYNA and XA have been shown to be the most efficient free radical-scavengers among Trp and Trp metabolites [56], which may due to the similar structures of these two molecules [43]. The antioxidant activities of XA in vitro have been shown [7,64].

Zsizsik and Hardeland showed that XA inhibited iron-mediated LP and copper oxidation in low-density lipoprotein, which demonstrated that the antioxidant property of XA is related to its metal-chelating activity. XA could also enhance the regeneration of reduced GSH by stimulating the supply of NADPH [77]. Moreover, XA could scavenge ^•^OH in a hematoxylin auto-oxidation system [59]; among the antioxidants tested, XA was an effective antioxidant, ranking only second to melatonin [78]; XA could accelerate oxygen consumption, and revealed its antioxidant properties [79].

XA has been shown to be a scavenger of peroxyl radicals in vitro, but its function as an antioxidant in vivo has been less studied. Recently, large amounts of XA have been detected in the midgut of *Aedes aegypti*, and XA has been shown to be an antioxidant based on heme or iron in this tissue [80]. The antioxidant properties of XA in vivo need to be explored further.

On the contrary, sometimes, the pro-oxidant action of XA is the result of its function of chelating. Previous studies found that XA could stimulated the auto-oxidation of ferrous ion with 8-hydroxyl group. Furthermore, the formation of metal-chelate complex (containing XA) is responsible for the generation of ROS [81,82].

### 4.7. Indoleamine 2,3-Dioxygenase

IDO catalyzes the cleavage of the pyrrole ring of Trp to form *N*-formyl-kynurenine. Apart from its catalytic function, the antioxidant properties of IDO have also been proposed. IDO can scavenge superoxide radicals directly [7,83], and IDO is considered to be a genuine antioxidant enzyme. During catalysis, IDO uses one O_2_^•−^ and its affinity for O_2_^•−^ is greater than that of SOD [7]. Because of the important role in the clearance of O_2_^•−^ by IDO, the decreased expression of IDO is responsible for increasing OS in placentas [84]. In the human eye, free Trp can be degraded by IDO, which could be an antioxidant mechanism in the eye [83]. In the epididymis tissue of mice, IDO has been suggested to be an essential antioxidant enzyme for protection of the epithelia from the damage caused by generated ROS [41]. The low level of IDO in human placentas has been assessed; decreased activity of IDO is associated with OS in the preeclamptic placenta [85]. During pregnancy, IDO may reduce free-radical damage on the vascular endothelium of fetal and maternal vessels [84].

In the same time, the interaction between IDO and nitric oxide (NO) should be given attention. NO can be produced by inducible isoform of NO synthase (iNOS) which was expressed in macrophages from some mouse strains, but not from human, pigs, rabbits and goats [86,87]. In the immune system, NO, similar to IDO, could induce by IFN-γ and LPS in mice [88]. Besides playing an immunoregulatory role in human cells, NO could interact with Trp metabolism in some ways. The regulatory crosstalk between NO production and IDO is complicated. Firstly, NO was proven to be an important regulator of IDO [89]. In vivo studies indicate that NO can inhibit IDO catalytic activity by directly interacting [90] or by stimulating IDO degradation through the proteasome pathway [91]. Secondly, 3HAA, a Trp metabolite, could inhibit the expression and catalytic activity of iNOS [92]. On the contrary, another Trp metabolite, picolinic acid, could induce IFN-γ-dependent iNOS expression [93]. The coherent molecular mechanisms underlying the NO-related inhibition of IDO remain unknown.

How can the underlying influence of OS by interaction between IDO and NO be explained? The antioxidant properties of IDO were proven, so the inhibiting of IDO by NO may restrict the antioxidant properties and induce increased free radicals. NO, a free radical, owns ability to scavenge other free radicals, such as H_2_O_2_ and O_2_, and, is placed in a pivotal regulatory position [94]. In the properties of scavenge free radicals, NO may play as a competitive inhibitor of IDO. In addition, in vitro study showed that the NO-dependent inhibition mechanisms of hIDO (human recombinant IDO) can be regulated by some cellular factors, such as pH, redox environment, and, NO and Trp abundance [94]. Thus, to assess how these factors affect NO-dependent IDO activity under physiological conditions is useful.

Moreover, the differences in macrophage NO and IDO production and regulation among different species should be concerned [95,96,97]. In previous studies, fundamental differences of NO synthase (NOS) activity between macrophages from mice and humans were described in the response of macrophages to IFN-γ [97]. Murine macrophages could produce abundant NO and l-citrulline from l-arginine via induction of the iNOS. However, macrophages from human and many other animal species, such as rabbits, or goats, do not have NOS activity [98,99]. Conversely, IFN-γ treatment in macrophages strongly induces IDO in human but not in murine cells [100]. These should be deserved special attentions when investigating the underlying influence of OS by interaction between IDO and NO in immune system in different species.

## 5. Trp Metabolism during Normal Pregnancy

During pregnancy, the demand for Trp in maternal and embryonic bodies increases. In addition, Trp availability in plasma is increased throughout pregnancy, manifesting as decreased concentrations of Trp and increased concentrations of kynurenine in plasma [101]. In humans and rats, the total Trp concentration in plasma decreases during normal pregnancy [102,103]. The higher Trp concentrations in umbilical-cord blood reveal increased transport and tissue uptake of Trp and increased Trp use for various physiologic and host-defense functions during pregnancy [102,104]. Immune factors, an altered hormonal environment, and OS have been suggested to induce changes in the metabolism and use of Trp during pregnancy [105,106].

During pregnancy, beside the increased requirements for protein synthesis, increased Trp metabolism via the KP is a host defense response and causes increased levels of kynurenine metabolites, which are required for the implantation, growth and development of the embryo and, possibly, also as a regulatory mechanism of the cellular redox state [104,107]. In another respect, 5-HT and NAD may affect energy metabolism during gestation. Increased production of 5-HT is crucial for increasing maternal insulin secretion, which is needed to overcome the insulin resistance associated with gestation [108]. The NAD pathway is also enhanced in pregnant rats and women [109]. In tissues, the regulation of gene expression and endocrine signaling by the NAD-dependent protein deacetylase sirtuin may be affected during gestation [110]. 

IDO, the first enzyme of the extrahepatic KP, is activated in pregnancy and its expression is correlated with placenta development [111,112]. Interestingly, IDO activation and decreased levels of Trp are considered to be related to immune activation during pregnancy [112]. One study has suggested that cytokine-induced IDO activation is involved in Trp degradation [112]. The cytokine IFN-γ, expression of which is induced by infection or inflammation in mammals, is activated powerfully by IDO in various cells and tissues [112]. Some researchers have suggested that this activation is related to the suppression of infection through limitation of Trp availability [107]. This action restricts the growth of pathogens by preventing protein synthesis and cell division, thereby participating in host defense [113]. However, another viewpoint regarding IDO activation is that IDO is not related to limitation of Trp as an essential amino acid in infection, but is more relevant to stress responses and the production of kynurenine metabolites, which can regulate the activities of antigen-presenting cells. This hypothesis also suggests that Trp depletion is not a defense response, but is instead a consequence of Trp consumption [104]. Thus, interference in IDO activity, IDO reactants, or IDO products may represent novel therapeutic approaches for immune disorders during pregnancy.

### 5.1. Variation of Trp Metabolism in Different Stages of Pregnancy

During pregnancy, the concentration of Trp and some Trp metabolites varies over time. In the early and middle stages of pregnancy, levels of free and total Trp are increased in proportion with TDO inhibition by progesterone and estrogen. In the late stage, levels of free Trp are increased markedly, whereas those of total Trp are decreased, due to increased uptake in tissues and the rapid equilibration between free and albumin-bound Trp [102]. Kynurenine levels increase simultaneously with decreases in Trp levels during gestation [103]. Serum concentrations of kynurenine are lower in non-pregnant sheep than those in pregnant sheep. Correlation between the kynurenine:Trp ratio and gestation days has been observed (*r* = 0.714, *p* < 0.001) [103,114]. The highest concentration of serum kynurenine has been observed during the third trimester of pregnancy. Levels of another Trp metabolite, KYNA, increase significantly in the second trimester of pregnancy (fold change = 3.96) compared with the first trimester, a result that is in accordance with other plasma metabolomic studies [115,116]. In addition, levels of indole, the main Trp metabolite in bacteria, increase 2.03-fold during the third trimester, suggesting a significant increase in Trp degradation by bacteria in late pregnancy [115]. Levels of Trp metabolites in the umbilical vein and fetal artery have been found to be significantly higher than those in the maternal vein [117]. During the postpartum stage, Trp concentrations tend to increase and normalize, but kynurenine concentrations increase even further [103]. In addition, increased kynurenine concentrations have been suggested to be related to postpartum mood disturbances [118].

### 5.2. Trp Metabolism in the Placenta

In the placenta, Trp-degrading enzymes induce the exhaustion of Trp levels and the formation of bioactive Trp metabolites at and near the sites of catabolism. The latter are essential for the establishment and maintenance of fetal–maternal immune tolerance and may also affect placental circulation and growth, as well as modulate local antimicrobial activity.

Expression of many key KP enzymes, such as IDO and TDO [119,120], has been detected in the placenta, and increases with placental development during healthy pregnancies [105]. These actions induce decreased concentrations of Trp and increased concentrations of KYN metabolites in the blood of pregnant women. These KP enzymes in the placenta catalyze Trp degradation, and can produce Trp metabolites, including KYN, KYNA, 3HAA, picolinic acid and QUIN; then, QUIN can be degraded to nicotinic acid [113]. All of these Trp metabolites have also been detected in umbilical-cord blood [113,120]. Furthermore, expression of several KP enzymes in the placenta is up-regulated in response to infectious conditions [119]; and placental production of kynurenine and QUIN increase accordingly upon exposure to inflammation provoked by infection in women in late pregnancy [120]. Throughout pregnancy, KP activity in the placenta is susceptive to infection and inflammation, confirming that the KP and its enzymes in the placenta may play an important part in the maternal and placental response to infection [84,113].

Second, Tph1 is also highly expressed in the placenta of several species [121], especially in mice. 5-HT synthesis in the placenta is important for early brain development in the fetus [122]. Tph1 may also contribute to Trp deprivation in the placenta, and can regulate immune tolerance and inflammation [123].

## 6. Trp Metabolism in Abnormal Pregnancies

Trp and Trp metabolism may also have significant roles in the processes of abnormal pregnancies. Studies have suggested that abnormal pregnancies may be associated with excessive Trp metabolism, which can induce pathologic immunosuppression by excessive production of kynurenine metabolites [104]. During a normal pregnancy, regulation of the level and metabolism of Trp is beneficial for establishing a balance between the needs of Trp by the fetus and safeguarding the fetus from maternal rejection. Destruction of this balance may induce the processes of abnormal pregnancies. There are two possible scenarios for this imbalance. First, if there is a severe deficiency of Trp and decreased production of immunosuppressing KP metabolites, the essential nutrient needs of Trp cannot be met, which undermine the suppression of T-cell responses. Second, an excessive amount of Trp is likely to reverse the immunosuppression by KP metabolites, thereby causing pregnancy complications [124]. Feeding of a diet high (5%) in Trp induced decreased placenta, fetal-body and pup weights, and increased the mortality of mice pups [125]. In preeclampsia, reduced expression of IDO1 and Trp-degrading activity in the placenta, and the correlation between reduced placental Trp-degrading activity and disease severity, have been reported [121].

During human pregnancies, increased OS has been reported to be associated with preterm labor, fetal intrauterine growth restriction (IUGR), preeclampsia (PE), recurrent miscarriage (RM) and other pregnancy complications [3,126].

### 6.1. Intrauterine Growth Restriction

Intrauterine growth restriction (IUGR), defined as weight below the 10th percentile for the gestational age, is a harmful pregnancy disorder, which can induce adverse consequences for fetus and infant [110]. Many factors may cause IUGR. A common cause of IUGR is the abnormal placental function. Impaired placental function, such as reduced activity of placental nutrient or oxygen transporters, conduces to the etiology of IUGR [84]. OS also plays a role in IUGR [127]. In patients with IUGR, the total antioxidative activity in serum is depressed and OS is increased [127,128], the processes of LP are enhanced and LP is increased [128,129,130]. The concentrations of OS parameters (maternal plasma levels of SOD, GSH-Px, and MDA) were significantly higher in IUGR patients than in normal pregnancy [127,130]. 

In IUGR, maternal–fetal Trp transfer is unaffected, but 5-HT production and activity of KP are changed [84,110]. Maternal 5-HT production decreases in IUGR, which may impair maternal insulin secretion, and then alter carbohydrate metabolism, whereas fetal 5-HT synthesis remain unaffected [110]. Furthermore, activity of KP, reflecting as the kynurenine or downstream metabolites production, is decreased in IUGR due to the influence of enzyme activities within the pathway by placental or fetal hypoxia. For details, IDO and some kynurenine enzymes are lower expressed in the IUGR placentas caused by changes of oxygen environment [131,132,133]. The decreased IDO expression might also be associated with the heightened placental inflammatory status in IUGR due to decreased free radical scavenging activity mediated by IDO in feto-placental tissues and uterus [84]. At the same time, the conversion to QUIN was impaired, and indirectly induced the decrease of NAD production and activity of sirtuins in IUGR [110].

### 6.2. Preeclampsia

Preeclampsia (PE) is a common pregnancy complication characterized as maternal hypertension, proteinuria and inappropriate inflammatory response, which can cause maternal and fetal morbidity and mortality [134,135]. The progression of PE involves placental ischemia, which may cause fetal hypoxia and acidosis, and subsequently leads to poor outcomes for both mother and baby [136,137]. In addition, the breakdown of immune tolerance, hypoxia, OS, excessive inflammation are suggested to be associated with the etiology of PE [85,138]. 

OS is involved in the pathophysiology and development of PE [139,140,141]. In PE, increased levels of OS were reported to be associated with ischemia-reperfusion injury and vascular dysfunction in placenta [142,143]. On the other side, increased antioxidants could decrease the risk of PE [141].

IDO might play a crucial role in the clinical features of PE [134]. Reduced IDO expression and attenuated Trp catabolism in placenta were detected in the PE group [135,144]. The IDO expression in endothelial cells was down-regulated in PE placentas, which might be related to shallow placentation, an incentive of PE [145,146,147]. In addition, decreased IDO expression was found to be associated with the severity of PE, containing severe maternal hypertension and proteinuria, dysregulation of the inflammatory response [134,135]. In pregnant mice, pharmacological inhibition of IDO induced the developing of maternal hypertension and proteinuria, and impaired local placental circulation, which have yielded features similar to human PE [148,149]. In another aspect, lacking of IDO function may be a risk factor for PE [149]. In pregnant IDO knock-out mice, PE phenotypes, such as proteinuria, pregnancy-specific endothelial dysfunction, IUGR, and mildly elevated blood pressure were detected [102,149].

IDO may also be associated with the oxidative damage on the placental endothelium of PE [139,142,150]. IDO catabolizes Trp by utilizing O_2_^•−^ radicals [151]. Decreased activity of IDO in PE may induce the reduced clearance of O_2_^•−^, thus contributing to the oxidative damage in PE [149]. Base on mentioned, reasonable regulation of IDO activity, IDO reactants, or IDO products may represent novel approaches for PE prevention and therapy.

In addition, the excretion of XA is affected by PE. After ingestion of Trp, the amounts of excreted XA in urine in PE and eclamptic patients are much larger than that in normal non-pregnant and normal pregnant women under the same conditions [152]. The detection of XA excretion may have important value in early diagnosis of PE or eclampsia.

### 6.3. Recurrent Miscarriage

Recurrent miscarriage (RM), defined as at least two consecutive spontaneous pregnancy losses before 20 weeks gestation, affects up to 5% of women around the world. Some known factors, including infection, chromosomal abnormalities and uterine abnormalities, were proven to cause RM, but approximate 50% cases were unexplained [145]. Immunological dysfunction and OS were suggested to be the cause of the unexplained proportion [146,153]. A disruption of the balance between the pro-oxidant and antioxidant may occur in patients with RM. In patients with unexplained RM, decreased concentrations of antioxidants (plasma vitamin C, vitamin E, β-carotene and erythrocyte GSH), elevated plasma levels of lipid peroxides, reflected the enhancement of OS [154,155]. In addition, increased generation of ROS was detected in leukocytes from recurrent miscarriage (RM) patients compared with healthy women [156].

IDO is important in maintaining maternal-fetal tolerance and immunological tolerance, and also has antioxidant property. Inhibition of IDO induced abortion in pregnant mice. In addition, IDO blockage caused an inflammatory reaction in the maternal-fetal interface [145]. In RM patients, the protein and mRNA levels of IDO in placental trophoblasts were significantly lower than those in normal pregnancies, which were consistent with other reports [157,158]. In another report, the proportion of IDO-positive cells was reduced in decidua from 30% RM patients [159]. Insufficient IDO leads to decreased proliferative and migratory capacity of trophoblast cells via suppressing signal transducer and activator of transcription 3 (STAT3) phosphorylation and matrix metallopeptidase 9 (MMP-9) expression in RM patients [145]. Conversely, the overexpression of IDO can promote trophoblast cell proliferation and migration. For these, the differential expression of IDO and dysfunction on activation of IDO in placenta may play an important role in the disease progress of RM [160].

### 6.4. Offspring Atopic Dermatitis (AD)

AD, also known as atopic eczema, is a type of inflammatory skin disease with unknown etiology. AD may paroxysm at any age, but most often in infancy and childhood, and becomes more serious through time [161]. The pathogenesis of AD is associated with OS [161]. Increased OS parameters (MDA, 8-hydroxy-2′-deoxyguanosine (8-OHdG)), decreased non-enzymatic antioxidants (GSH, and Vitamins A, E and C) were detected in AD patients than in healthy controls, OS was suggested to be contributing factor in the pathogenesis of childhood AD [161]. A significant positive correlation between levels of OS and the severity of AD was confirmed [162,163].

Some environmental factors during pregnancy are associated with the increased risk of AD, including maternal nutrition ingestion [164]. During pregnancy, Trp metabolism and metabolites were proved to be associated with the occurrence of offspring AD. Later pregnancy, higher maternal concentrations of nicotinamide and anthranilic acid were associated with a lower risk of offspring AD at 12 months old [164]. More active Trp metabolism is suggested to be beneficial to ameliorate symptoms of AD. IDO and kynureninase are higher expressed in skin lesions compared with the uninvolved skin of patients with AD, which might be partially due to stimulation of Fcε *R*1 expressing on monocytes with IgE and antigen [165]. Fcε *R*1 can activate the production of IDO by monocytes and contribute to self-limitation of immune responses, which plays an essential role in allergic diseases containing AD [166].

## 7. Factors Influencing Trp Metabolism and OS during Pregnancy

**Figure 2:**
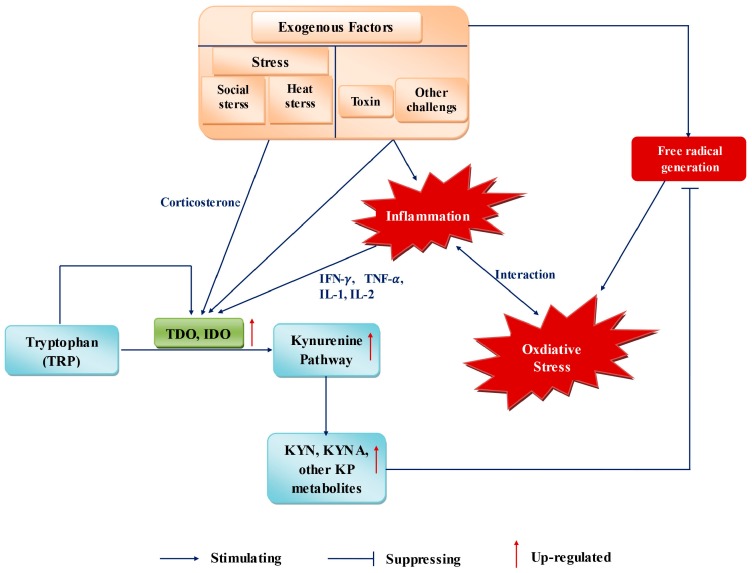
Kynurenine pathway acts as a regulator of antioxidant responses and support complementary antioxidant capabilities when bodies influenced by some exogenous factors. TDO, tryptophan 2,3-dioxygenase; IDO, indoleamine 2,3-dioxygenase; IFN-γ, interferon-γ; IFN-α, interferon-α; IL-1, interleukin-1 family; IL-2, interleukin-2; KYN, kynurenine; KYNA, kynurenic acid; KP, kynurenine pathway.

During pregnancy, numerous conditions, such as inflammation, social stress, heat stress, and toxin challenge, can influence the requirement and metabolism of Trp, as well as OS in the organism (Figure 2) [167].

Stress responses are associated with Trp metabolism. “Stress” comprises the causes and physiologic reaction of the organism towards internal or external changes. The latter can be sanitary conditions, feeding density, and thermal environment. Internal changes can be due to inflammation, anxiety or depression. For example, if the dietary Trp supply increases after an inflammatory response, the conversion of Trp to kynurenine is increased [168]. The Trp requirement of pigs can be affected if they are housed in poor sanitary environments [169]. In different species, dietary Trp is associated with aggressive behavior [170]. In stressed pigs, a large dietary supply of Trp can have a positive impact on meat quality [167].

### 7.1. Inflammation

Inflammation is the physiologic response of tissues to harmful stimuli. In farms, infectious diseases and other harmful stimuli may induce the inflammatory status of breeding animals. IDO1 and related Trp catabolism have been identified as factors endowed with immunomodulatory effects [171].

Pregnancy can be characterized as a physiologic systemic inflammatory response, the stages of which are closely related to OS. The concentrations of inflammatory reactants (i.e., fibrinogen, plasminogen activator inhibitor-1, and ceruloplasmin) are increased during gestation [172]. Increased concentrations of interleukin (IL)-12 are related to activation of the innate immune system during pregnancy [172]. Abnormal pregnancies and related complications are also associated with inflammation. Intrauterine infection and inflammation are important causes for preterm parturition, which can lead perinatal morbidity and mortality [172]. Preeclampsia, another cause of maternal mortality, is characterized by a more intense systemic inflammatory response than that observed in normal pregnancy [135]. Pro-inflammatory cytokines, including tumor necrosis factor-α and IL-1-β, are associated with the pathogenesis of preterm parturition and preeclampsia [173,174]. In addition, inflammatory processes have been reported to play a major part in spontaneous abortion [175].

The interrelationship between inflammation and Trp metabolism has been studied in pigs, mice and humans. Trp catabolism is increased under IDO activation in piglets, which suffer from chronic lung inflammation [176,177,178,179]. Inflammation affects Trp availability and the requirement for growth accordingly after modification of Trp metabolism in piglets [179]. In human dermal fibroblasts, IDO expression is increased after treatment with the pro-inflammatory cytokine IFN-γ, as are KYNA levels [180]. In elderly persons with chronic low-grade inflammation, decreased Trp levels and increased kynurenine concentrations are associated with increased inflammation [181]. Kudo and colleagues found, in normal pregnancies, an increased kynurenine:Trp ratio that was similar to that of disorders characterized by systemic inflammation; this finding could be attributed to the IDO activity of circulating leukocytes [135].

The cases described above could be explained by the fact that IDO activation induces an increase in Trp catabolism through the KP during inflammation. After feeding experiments, the inflammatory response is weakened if pigs are fed with an adequate amount of Trp [167]. Another explanation could be that upregulation of IDO expression and increased Trp catabolism might be a promising therapy to alleviate inflammation [182,183]. Consequently, Trp catabolism through the IDO–KP pathway has been proposed as a potential therapeutic target for controlling inflammation in normal pregnancy or related complications [184]. 

During inflammation, oxidative damage to host tissue and cells cannot be ignored. With regard to the relationship between inflammation, OS and Trp metabolism, we support the proposal that induction of Trp degradation along the IDO-KP helps to meet the requirement for antioxidant defenses that attenuate/prevent inadvertent oxidative damage during inflammation [185]. As has been reported, the effects of inflammation on IDO activity and Trp catabolism are decreased by antioxidant addition, which validates this proposal indirectly [179].

### 7.2. Social Stress

In intensive farming, many factors, such as regrouping, aggressive behaviors, and high feeding density can increases social stress, OS, and influence productive performance. Regrouping may increase social stress and aggression, which are proven to be associated with OS [16]. Mice that were exposed to restraint environment had increased LP level [186].

Social stress was proved to be associated with Trp metabolism in organisms. During pregnancy, restraint stress can induce the increase of some Trp metabolites along KP in maternal mouse and fetus [187]. An acute stress caused by restraint during the late gestational period can induce significant elevations of KYNA levels in the maternal plasma, placenta, and fetal brain, and raised levels of Trp and kynurenine in placenta, fetal plasma, and fetal brain [187]. The link between stress and KP might be mediated by corticosterone, which can directly activate TDO, that catalyzes the conversion of Trp to formylkynurenine. For this, stress indirectly activates TDO, and induces increase of kynurenine and downstream KP metabolites concentration in the plasm and periphery.

Aggression may be related to the reduction of plasma Trp [188]. In nursery piglets, weaned piglets, male mice, chick and rainbow trout (*Oncorhynchus mykiss*), appropriate dietary l-Trp supplementation can suppress aggressive behavior [170,189,190,191,192]. However, in other studies, high or surplus dietary l-Trp additions did not effectively reduce aggression and associated stress in young growing pigs or sows [193,194]. Thus, suggested that the addition amount of Trp could influence the suppression of aggression in pigs.

High feeding density is another factor causing stress, which can negatively affect broiler, pigs and others [195,196,197]. Negative effects can be ascribed to the generated stress which may induce increase of ROS or attenuated antioxidant status [198]. Dietary Trp supplementation can alleviate stress and OS caused by high feeding density. Significant interaction between stress and broiler stocking density was observed by plasma levels of cholesterol which is a stress indicator [199]. Under high feeding density, dietary Trp supplementation could alleviate stress and improve antioxidant capacity, growth performance and meat quality of duck [200]. Short term supplementation of l-Trp in pigs improved growth performance and reduced stress hormone during period of social-mixing stress that caused by animal regrouping [201].

### 7.3. Heat Stress (HS)

In livestock production, HS leads to poor growth performance and some other negative effects. HS was proven to disrupt redox balance, increase OS and induce oxidative injury [202,203,204,205], and OS was suggested to be one of major stress responses caused by HS [11,205]. 

HS was proven to influence OS in mice and sows during pregnancy [11]. Under high temperature environment, antioxidant capacity of mice decreases, which induce increased production of free radicals and oxidative damage during late gestation and lactation [206,207]. Under HS, OS and oxidative damages became worse, induced by increased ROS production, LP, protein oxidation, oxidative DNA damage and higher lipid and protein damage during late gestation and lactation compared with the sows under moderate temperature environment [11,206]. In addition, during the period of embryonic implantation, increased OS may induce increased embryonic death for sows under HS [208].

Under HS, the increased OS and increased oxidative damage to lipid, protein, and DNA, can reduce reproductive performance and longevity of sows [11,208], manifesting as decreased number of piglets born alive, litter weight at birth, piglets per litter on d 18 of lactation. Litter weight gain and litter size were negatively correlated with increased plasma concentrations of protein carbonyls, MDA, and 8-OHdG in sows [11].

The relation between HS and Trp metabolism has been detected in animals [209,210,211]. In rats, HS stimulated the increase of Trp pyrrolase in liver during 3 and 48 h exposures [209]. The concentration of Trp in liver was unchanged, but Trp in the plasma was reduced by half after 8 h exposure to HS and returned to normal by 46 h [209]. In broiler chickens, HS significantly increased plasma Trp concentrations at 30 min exposure [210,211]. The discrepancy of variable trend of Trp concentration between rat and chicken had been reported, the possible reasons may be the choose of different check point in time, or due to the inner difference among species. For information on the relationships among HS, OS, and Trp metabolism suggesting that, under HS, Trp and its metabolism may play a role in regulating OS, changes of Trp and its metabolite concentrations might be used to attenuate heat effects.

### 7.4. Toxin Challenge

Toxins can also cause OS in organisms. Study of the association between toxins and Trp metabolism has been undertaken. In human peripheral blood mononuclear cells, streptococcal erythrogenic toxins were able to induce Trp degradation [212]. LPS, a known endotoxin, can enhance IDO activity and regulate stress responses through mediating Trp metabolism along KP [213]. In pigs, mycotoxin (aflatoxin and deoxynivalenol) can reduce feed taking, increase systemic inflammation and OS even at low level [214,215]. In weaned pigs, diquat-induced OS stimulated Trp degradation via KP through upregulation of TDO expression in the liver, decreased the Trp concentration in serum, and then reduced growth [216]. Following, increasing dietary Trp could attenuate the liver OS induced by diquat via enhancing the antioxidant capacity of weaned piglets [217]. Hence, we suggest that adjustment of dietary Trp supplementation may be a practical way to attenuate the OS caused by some toxins.

## 8. Potential Antioxidants of Trp Metabolism during Pregnancy

Here, we have discussed OS and variations in Trp metabolism during pregnancy. In normal or abnormal pregnancies, the higher metabolic demand and increased oxygen consumption leads to increased production of reactive species (ROS, RNS and others) and reduced antioxidant capacity, which induces OS. Especially in abnormal pregnancies, OS and oxidative damage to the body are enhanced, and are associated with physiologic disorders and pregnancy-associated diseases such as fetal intrauterine growth restriction, preeclampsia, and recurrent miscarriage. In normal or abnormal pregnancies, the metabolism and use of Trp have been shown to change as pregnancy progresses.

Direct evidence to elucidate the relationship between OS and variations in Trp metabolism during pregnancy is lacking. However, the underlying correlation or relationship between these two factors may be presented from available studies. For instance, during the late gestational period, OS is increased and the oxidative damage to protein and DNA is increased further. Simultaneously, the level of total Trp is decreased and the kynurenine level is increased, and the serum concentration of kynurenine is highest during the third trimester of pregnancy. In addition, a correlation between the Trp:kynurenine ratio and gestation days is observed. Though there is some evidence on the correlation between OS and variations in Trp metabolism, a single unifying theory that can show the variations in Trp metabolism to be a cause or an effect of OS during pregnancy is lacking.

When considering a new strategy to relieve OS during gestation through controlling dietary levels of Trp or regulating Trp metabolism, variations in OS and Trp metabolism during different stages of pregnancy should be evaluated. Simultaneously, because nutrient requirements are affected by the different stages of gestation, Trp should be assessed as a specific antioxidant and a nutrient in the body, with a focus on the complex composite effects of its different features rather than a single feature. As an antioxidant or precursor, the dietary Trp concentration should be noted at the time of a relevant pro-oxidant challenge and be re-evaluated for its adequacy in diets to prevent excessive OS during late gestation and lactation. Learning from past failures using traditional antioxidants, we propose that supplementation with Trp as an antioxidant should be before placentation in the early second trimester to prevent OS.

Besides, during pregnancy, immunoregulatory aspect of Trp-degrading is extremely important for maternal-fetal immune tolerance. IDO-induced Trp metabolites were suggested to mediate tolerance in pregnancy [218]. IDO expresses in placental trophoblast cells, decidual cells, and decidual stromal cells, which may prevent exclusive attack by the maternal immune system [218,219]. In addition, Trp metabolites may act as key regulators of immune cell in abnormal pregnancies [104]. During pregnancy, excessive supply of Trp has been considered to be an important contributing factor in PE [166]. Decreased placental, foetal body and pup weight, and increased newborn mortality were observed in pregnant mice fed a high (5%) Trp diet [125]. A possible mechanism is that excessive Trp supply reverses the immunosuppression by kynurenine metabolites or IDO, resulting in pregnancy toxaemia or other pregnancy complications [219]. For these, inappropriate manipulation of Trp metabolism that whether disorganize the immunoregulatory properties or break redox properties of Trp metabolism could induce undesired side effects. These two impact factors should be considered in further studies.

## 9. Summary and Future Perspectives

Strong evidence is accumulating in support of the use of Trp and its metabolites based on its antioxidant properties. Investigating the concept of using Trp and its metabolites will be important in future studies on pregnancy.

The potential for use of Trp as its antioxidant properties in pregnancy can be observed in four main aspects. First, a dynamic change in Trp metabolism is an accommodation mechanism in response to OS in organisms. The increased Trp metabolism and Trp metabolites help the body to cope with the OS caused by increased basal oxygen consumption during pregnancy. Second, though Trp and its metabolites have antioxidant properties, a continuously increased supply of Trp is impossible, and may influence the maternal and fetal health, as well as cause long-term and irreversible effects. Third, according to the variations of Trp metabolism at different stages during pregnancy, control of Trp supplementation and regulation of Trp metabolism may be a valid approach to control and attenuate OS. Finally, by controlling the dietary level of Trp or regulating Trp metabolism, OS-mediated injury could be alleviated in pregnancy-associated disorders caused by oxidative damage.

Nevertheless, three important issues must be addressed in future studies. First, OS and Trp metabolism at different pregnancy stages, and the relationship between them, must be investigated to obtain direct evidence of the interactions between them. Second, Trp use along different pathways during pregnancy must be assessed through animal experiments. In this way, the amount of Trp used for a single pathway or physiologic process can be evaluated. Finally, assessment of the optimum physiologic concentration of Trp at different pregnancy stages or in pregnancy-associated disorders must be assessed in animal experiments. This can guide the Trp supply to prevent such disorders during pregnancy.

## Abbreviations

Trp	Tryptophan
OS	Oxidative Stress
NAD	Nicotinamide Adenine Dinucleotide
NADP	Nicotinamide Adenine Dinucleotide Phosphate
ROS	Reactive Oxygen Species
RNS	Reactive Nitrogen Species
ATP	Adenosine Triphosphate
MDA	Malonaldehyde
pO2	Pressure of Oxygen
Hb	Hemoglobin
KYN	Kynurenine
KP	Kynurenine Pathway
TDO	Tryptophan 2,3-dioxygenase
IDO	Indoleamine 2,3-dioxygenase
KYNA	Kynurenic acid
3HAA	3-Hydroxyanthranilic acid
3-HK	3-Hydroxykynurenine
XA	Xanthurenic Acid
QUIN	Quinolinic Acid
SOD	Superoxide Dismutase
KATs	Kynurenine Aminotransferases
HOCl	Hypochlorous acid
GSH	Glutathione
GST	Glutathione S-transferase
NO	Nitric Oxide
LP	Lipid Peroxidation
IFN-γ	Interferon-γ
LPS	Lipopolysaccharides
iNOS	Inducible Nitric Oxide Synthase
HO-1,	Hemeoxygenase-1
Nrf2	NF-E2 Related Factor 2
5-HT	5-Hydroxytryptamine
AA	Anthranilic Acid
Tph1	Trp Hydroxylase-1
IUGR	Intrauterine Growth Restriction
PE	Preeclampsia
RM	Recurrent Miscarriage
STAT3	Signal transducer and activator of transcription 3
MMP-9	Matrix Metallopeptidase 9
IL	Interleukin
TNF-α	Tumor necrosis factor-α
HS	Heat stress
